# Monitoring and Human Health Assessment of Trace Elements in Fresh and Dry Fish From Lake Kariba, Siavonga, Zambia

**DOI:** 10.1007/s12011-026-05263-7

**Published:** 2026-08-05

**Authors:** Phaphedi M Poopedi, Sinegugu Khulu, Kawawa Banda, Heidi Richards, Mokgaetji Monyai, Imasiku Nyambe, Yoshitaka Uchida, Luke Chimuka

**Affiliations:** 1 Department of Chemistry, Faculty of Science, Engineering and Agriculture, University of Venda, Private Bag X5050, Thohoyandou, 0950 South Africa; 2 Molecular Sciences Institute, School of Chemistry, University of Witwatersrand, Private Bag X3, Johannesburg, 2050 South Africa; 3 School of Education, University of Witwatersrand, 27 St. Andrews Rd, Parktown, Johannesburg, 2193 South Africa; 4 Integrated Water Resources Management Centre, School of Mines, University of Zambia, P.O. Box 32379, Lusaka, Zambia; 5 Research Faculty of Agriculture, Hokkaido University, Kita 9, Nishi 9, Kita-ku, Sapporo, 060-8589 Hokkaido Japan

**Keywords:** Health risk, Trace elements, Hazard Index, Lake Kariba, Fish

## Abstract

This study investigated the levels of trace elements in two fish species, Kariba bream (Oreochromis mortimeri) and Kapenta (Limnothrissa miodon) from Lake Kariba in two consecutive years, 2021 and 2022. Both Kariba Bream fresh/sun-dried muscle and sun-dried Kapenta tissues were digested using a microwave digestion system and analyzed using Inductively Coupled Plasma Mass Spectrometry. Mean concentration of trace elements between the first and second sampling differed significantly (*P* > 0.05). In the first sampling, concentrations of trace elements in sun-dried bream occurred in the order Fe > Al > Cr > Zn > Mn > As while Se, Cu, Cd, Pb and Hg were not detected. Sun-dried Kapenta displayed Al > Fe > Zn > Mn > Cr > Cu > As > Se with Cd, Pb and Hg not detected. Fresh bream showed Fe > Al > Zn > Cr > Mn > Cu > Se, with Pb, As, Cd and Hg not detected. In the second sampling, the order was, Fe > Al > Zn > Cr > Mn > Se > Cu > As in sun-dried bream; Fe > Zn > Mn > Al > Cr > Cu > Se > As in sun-dried Kapenta with Cd, Pb and Hg not detected in both. Fresh bream showed; Fe > Al > Zn > Mn > Cr > Se > Cu > As > Pb with Cd and Hg not detected. Notably, the concentrations of Fe, Mn and Cr in some samples exceeded the recommended limits by FAO/WHO. The THQ and HI for some elements were above the threshold value of 1, indicating potential risk of non-carcinogenic to human health for long-term consumption. These findings highlight the need for further investigation into post-fish handling, including land-use activities in and around the lake.

**Supplementary Information:**

The online version contains supplementary material available at https://doi.org/10.1007/s12011-026-05263-7.

## Introduction

The presence of pollutants in water and biota results from both natural and anthropogenic activities leading to environmental health issues [1, 2]. The diverse sources of these anthropogenic pollutants include industrial waste that includes mining, agricultural practices, poor functioning and sometimes unavailable sewage systems. These sources often have led to elevated levels of both organic and inorganic endocrine disrupting compounds (EDCs) in biota such as fish [3, 4]. These pollutants contaminate the water and surrounding ecosystem and bioaccumulate in fish [1, 2]. It is predicted that the quantities and toxicities of EDCs related pollutants are expected to increase in areas with high population growth and economic activities, further exacerbating these issues [5–7]. One of the common pollutants in marine and aquatic environments is trace elements (TEs), which have been detected in many water bodies such as surface waters at concentrations ranging from ng/L to µg/L, which over time lead to the contamination of biota [2, 6, 8]. Fish and sediments are amongst the mostly used critical bioindicators of longer-term pollution [6, 9, 10].

Fish contributes to the human diet as a source of protein. On the other hand, fish has the potential to bioaccumulate some TEs which are toxic to human health, especially at high concentrations in water bodies [11, 12]. Their toxicity occurs when the elements exceed their naturally occurring level in the environment which affects human and animal health [13]. Elements with no known biological functions such as Cd, Pb and Hg, can exhibit extreme toxicity even at traceable amounts [14, 15]. These are listed among the major trace elements of public health concern [16, 17].

Large water bodies like Lake Kariba are now of interest regarding their ecosystems’ health which can be linked to various activities occurring in and around the lake. Several studies focusing on TEs’ concentrations in fish and their potential health risk through dietary intake have been reported globally [8, 18, 19]. However, to the best of our knowledge, limited studies have been reported on the status of pollutants in Lake Kariba and very few focusing on the potential risk assessment of TEs on edible fish locally sold in the Zambian open markets [1, 2, 13, 20, 21]. A recent study by Simukoko and colleagues [13] compared the levels of TEs in the wild and farmed Kariba bream. The study found that the concentrations of TE’s were below the limits established by the World Health Organization Europe (WHO/EU), where essential elements were higher in farmed bream while non-essential elements were elevated in wild bream [13]. On the other hand, Miyanza and coworkers looked at concentrations of TEs in raw commonly used vegetables and fish sold in open markets of Lusaka, Kitwe, and Kabwe towns of Zambia [20]. However, these studies never looked at dry Kariba bream which is part of this work.

This study aimed to investigate the levels of TEs in two fish species, Kariba bream (*Oreochromis mortimeri)* and Kapenta (Limnothrissa miodon) harvested from the lake. The objective was to evaluate the distribution of trace elements in fresh bream, dry bream and Kapenta, specifically focusing on the assessment of health risk associated with TEs concentration in fresh and dry fish. This was performed to derive informative recommendations for the management of food safety and environmental pollution in the Lake.

## Materials and Methods

### Study area and Sampling

**Fig. 1:**
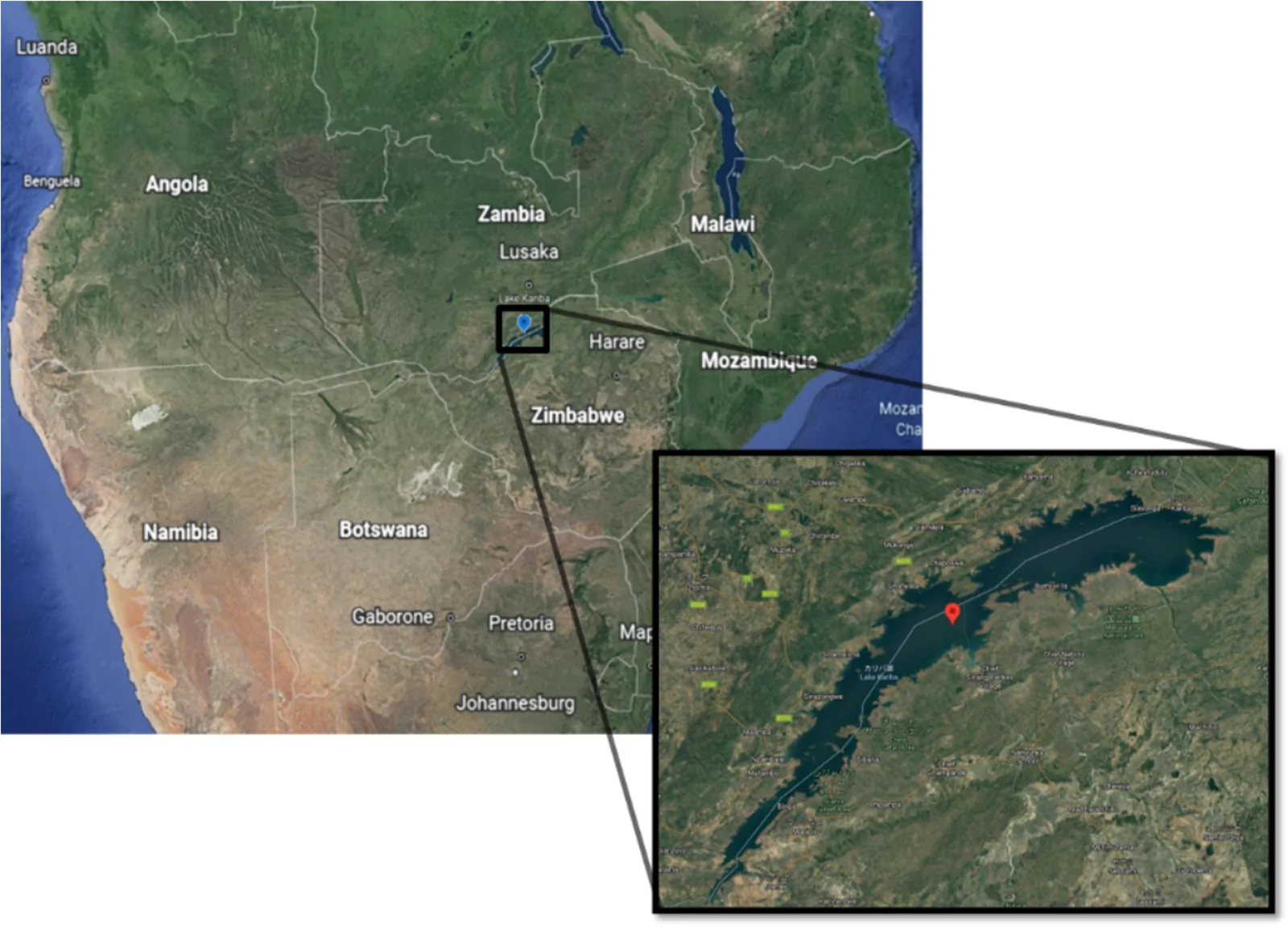
Map of the study area showing geographical location of Siavonga district along Lake Kariba

The study was conducted in Lake Kariba (Fig. 1), situated in the middle of the Zambezi River Basin in the southern province of Zambia, and is shared with Zimbabwe (− 17° S 28° E). Lake Kariba is currently the largest man-made reservoir in Africa by volume [21]. It is surrounded by three districts from the Zambian side, namely, Sinazongwe, Gwembe, and Siavonga each with a population density estimated at 98 246, 50 136 and 58 864, respectively [13]. The Lake is well known for Kapenta fishing [13, 22, 23]. Lake Kariba harbors the country’s largest aquaculture producing companies, mainly in cages based in Siavonga area of the lake [22].

Fish of interest, freshly and sun-dried Kariba bream and sun-dried Kapenta, were bought from the open market in Siavonga. Dry fish were stored in a cool, dry place. The fresh fish muscles were removed and wrapped in aluminum foil, then stored in zip-lock plastic bags. The muscles were then stored in the freezer before preparation and analysis for metals. Twenty-eight fresh and five sun-dried Kariba bream fish samples and sun-dried Kapenta fish batch samples were obtained in November 2021 during the first sampling. On the second sampling, four sun-dried Kapenta batches, six fresh Kariba breams, and five sun-dried Kariba breams were collected in August 2022. Targeted sampling sites were where most people purchase their foodstuffs, mostly markets that operate near Lake Kariba.

### Sample Preparation

Blank and recovery studies were conducted on commercially available fish samples bought from Johannesburg CBD. The muscles were transported into cooler boxes with ice blocks. At the lab, they were rinsed with distilled water and kept in the deep freezer for 72 h. From there, they were freeze-dried, followed by homogenizing into fine powder using a domestic blender and further ground to very fine particle sizes with a pestle and mortar. For the dry fish samples in each, a mass of 5 g of the sun-dried muscle sample was rinsed with distilled water and oven-dried at 40 ℃ until a constant weight was obtained, which was the total weight of the sample used for analysis. The dry fine powder samples were then stored in the PTFE tubes lined with parafilm and kept frozen until further analysis. The fish samples were prepared the same way.

### Reagents

All reagents and chemicals used for the analysis of trace elements were of analytical grade. 70% w/v nitric acid (HNO_3_) and 48% w/v hydrogen peroxide (H_2_O_2_) supra pure grade were purchased from Merck (Johannesburg, South Africa). Multi-element stock solution of 1000 mg L^− 1^ for ICP-MS was obtained from de Bruyn Spectroscopic Solution, South Africa.

### Instrumentation

All trace element concentration measurements were performed on an inductively coupled plasma mass spectrometry (ICP-MS) (Agilent 7700 Series, USA). Fish sample muscles were digested using the Multiwave Go Digestor, Anton Paar Southern Africa Pty Ltd (Johannesburg, South Africa). Deionized water was prepared from a Millipore water system (Massachusetts, USA). For all mass measurements, an analytical balance supplied by OHAUS (Johannesburg, South Africa) was used.

### Microwave Digestion of the Samples

For digestion of samples, a method adapted from Nuapia and coworkers [19] was employed. A mass of 0.25 ± 0.018 g of sample was weighed into digestion tubes (PTFE-TFM liners). A volume of 16 mL of HNO_3_ and 4 mL of H_2_O_2_ was added to the tubes. The tubes were sealed and placed into a microwave digestor rotor and digested at 180 °C. After digestion, the solution was transferred into 50 mL PTFE tubes, then diluted to the mark with distilled water. The solution was then filtered using 0.22 μm PVDF syringe filters and diluted before ICP-MS analysis. The same procedure was used for preparation of a blank to monitor contamination.

### Method Validation

#### Quality Control, Assurance and Recoveries

Quality control protocols were followed for all analyses to ensure reliability and accuracy of the results. Blanks were used to correct potential contamination. Calibration standards ranging from 5 to 100 µg L^− 1^ were prepared from the stock solution. Coefficient of determination (R^2^) was determined as a measure of linearity in the specified calibration range, and limits of detection (LOD) and limits of quantification (LOQ) were calculated based on the calibration curve method [24]. Furthermore, validation of the analytical method was conducted through spiking with 10 µg L^− 1^ of the target trace elements.

Table 1 displays the obtained method validation parameters. For all target TEs the obtained R^2^ values ranged from 0.9963 to 0.9999, showing good linearity across the calibration range. Good limits of detection (LODs) and limits of quantification (LOQs) values ranging from 0.84 to 11.74 and from 2.81 to 39.13 µg L^− 1^ were obtained, respectively. Moreover, good recoveries ranging from 90.66 to 107.8 were obtained showing good instrument performance. Good recoveries within the range of 70–120% are considered acceptable [25]. All samples were run in triplicate, and RSDs obtained were ≤ 1.75%, indicating repeatability and good precision for the analytical method.

**Table 1:** Method validation parameters

TE	Calibration Range (µg L^− 1^)	*R*^2^	LOD (µg L^− 1^)	LOQ (µg L^− 1^)	Mean Recovery (µg L^-1^)	% Recovery ± RSD
Al	5–100	0.9988	3.01	10.04	9.35	93.53 ± 0.34
Cr	5–100	0.9990	2.23	7.43	9.92	99.22 ± 1.72
Mn	5–100	0.9997	1.56	5.20	9.97	99.73 ± 1.72
Fe	5–100	0.9963	11.74	39.13	10.78	107.8 ± 1.49
Cu	5–100	0.9996	1.36	4.53	9.26	92.63 ± 1.22
Zn	5–100	0.9989	3.99	9.98	9.75	97.49 ± 1.75
As	5–100	0.9999	0.84	2.81	9.47	94.74 ± 1.59
Se	5–100	0.9993	1.60	5.33	10.72	107.2 ± 1.46
Cd	5–100	0.9996	1.23	4.09	9.38	93.76 ± 0.91
Pb	5–100	0.9993	1.60	5.34	9.07	90.66 ± 0.94
Hg	5–100	0.9997	1.07	3.58	9.77	97.73 ± 0.35

*n* = 3

### Statistical Analysis

Descriptive statistical analysis for the mean, standard deviation, and t-test for comparison studies was done using Microsoft Excel with ANOVA plugin and Origin Pro software for statistical significance (*P* < 0.05).

### Health Risk Assessment

The mean TEs concentrations in fish muscle were measured and compared to maximum limits (ML) set by WHO [26–28] for human consumption (Table 1). Human health risks associated with consumption of fish were assessed using estimated weekly intake (EWI), target hazard quotient (THQ), hazard index (HI) and Carcinogenic risk/cancer risks were calculated using Eqs. 3–6 [8, 13].

#### Estimated Weekly Intake (EWI)

The TEs concentrations and calculated estimated weekly intakes (EWI) were compared to provisional tolerable weekly intakes (PTWI) associated with human consumption set by WHO. EWI were calculated using Eq. 3. The estimated dietary exposure limits for meals according to the USEPA guideline were employed using the parameters used in literature [8, 13].

3 $$\:{\mathrm{E}\mathrm{W}\mathrm{I}}_{\mathrm{i}\mathrm{n}\mathrm{g}}=\:\frac{\mathrm{C}\:\times\:\:\mathrm{I}\mathrm{n}\mathrm{g}\mathrm{R}\:\times\:\:\mathrm{E}\mathrm{F}\:\times\:7}{\mathrm{B}\mathrm{W}\:}$$

Where, C = trace element concentration in fish (mg/kg), IngR = daily average ingestion rate per person (30.14 g/day), EF = Frequency of exposure (365 days/year), BW = Body weight (15 and 70 kg).

#### Target Hazard Quotient (THQ)

The target hazard quotient is the ratio between the estimated daily intake (EDI) and the oral reference dose (RfD, mg/kg bw/day). The target hazard quotient was calculated as demonstrated in Eq. 4

4 $$\:{\mathrm{T}\mathrm{H}\mathrm{Q}}_{\mathrm{c}\mathrm{h}\mathrm{i}\mathrm{l}\mathrm{d}/\mathrm{a}\mathrm{d}\mathrm{u}\mathrm{l}\mathrm{t}}\:=\:\frac{\mathrm{E}\mathrm{F}\:\times\:\:\mathrm{E}\mathrm{D}\:\times\:\:\mathrm{F}\mathrm{I}\mathrm{R}\:\times\:\mathrm{C}}{\mathrm{R}\mathrm{f}\mathrm{D}\times\:\:\mathrm{W}\mathrm{A}\mathrm{B}\:\times\:\:\mathrm{T}\mathrm{A}}\:\times\:\:{10}^{-3}$$

where E_F_ = Exposure frequency, E_D_ = exposure duration, F_IR_ = fish ingestion rate, C = mean metal concentration, RfD = oral reference dose, W_AB_ = average body weight of an adult and T_A_= average exposure time with noncarcinogenic effect (E_F_*E_D_).

RfD is an estimate of a daily oral exposure to a toxic substance that is likely to be without an appreciable risk of harmful effects during a lifetime [2, 13].

#### Hazard Index (HI)

The hazard index (HI) also referred to total target hazard quotient (TTHQ), is the sum of individual metal THQs. Equation 5 was used to calculate HI.

5 $$\:HI=\:\sum\:THQ$$

Where, ∑THQ is the sum of target hazard quotients (THQ) of all target TEs.

#### Carcinogenic Risk (CR)

Carcinogenic risk (CR) is estimated as the incremental probability of an individual to develop cancer over a lifetime, as a result of exposure to a potential carcinogen. The lifetime average daily dose to estimate the carcinogenic risk was calculated using Eq. 6

6 $$\:CR=EDI\:\times\:\:CSF$$

Where, EDI = Estimated daily intake (EWI/7), CSF = is the oral slope factor of a particular carcinogen in mg/kg per day, which are 1.5 (As), 6.3 (Cd), 0.5 (Cr) and 0.0085 (Pb) from the Integrated Risk Information System USEPA [29].

The total cancer risk (TCR) due to fish consumption was calculated as the sum of the individual cancer risks using Eq. 7 [13].

7 $$\:TCR\:=\:\sum\:_{\mathrm{i}=1}^{\mathrm{n}}\mathrm{C}\mathrm{R}\mathrm{i}$$

## Results and Discussion

### Total Mean Concentrations of Trace Elements

The results of the mean concentrations of the studied TEs in various fish are presented in Table 2 together with maximum permissible limits (MPL) as set by WHO/FAO. In the first sampling, their concentrations in fish samples were in a descending order with Fe > Al > Cr > Zn > Mn > As in sun-dried bream; Al > Fe > Zn > Mn > Cr > Cu > As > Se in sun-dried kapenta, and Fe > Al > Zn > Cr > Mn > Cu > Se in fresh bream. In the second sampling, the descending order was as follows: Fe > Al > Zn > Cr > Mn > Se > Cu > Pb > As in sun-dried bream; Al > Zn > Fe > Mn > Al > Cr > Cu > Se > As in sun-dried Kapenta; Fe > Al > Zn > Mn > Cr > Se in fresh bream, respectively. Hg and Cd were not present in all samples in both sampling periods, whilst Pb was only present in fresh bream at low concentration (Table 2). In sun-dried bream sampled in 2021, Cu and Se were not detected.

**Table 2:** Total mean TEs concentrations in fresh and sun-dried bream muscles and in sun-dried kapenta tissues (mg/kg ± SD) for the two sampling periods (2021 and 2022) and maximum permissible limits set by WHO/FAO

Sample	Essential TEs						Non-essential TEs				
	Cr	Cu	Fe	Mn	Se	Zn	Al	As	Cd	Hg	Pb
Sun-dried bream(n = 5) ^1^	15.47 ± 0.53	Nd	101.40 ± 22.69	6.50 ± 3.30	nd	10.06 ± 3.08	88.80 ± 38.21	0.03 ± 0.04	nd	nd	nd
Sun-dried kapenta (n = 5) ^1^	18.21 ± 1.07	1.09 ± 0.46	159.00 ± 8.79	39.28 ± 4.27	0.45 ± 0.37	91.62 ± 6.37	160.72 ± 132.60	0.49 ± 0.05	nd	nd	nd
Fresh bream (n = 28)^1^	19.53 ± 1.20	0.61 ± 0.93	94.92 ± 20.17	13.99 ± 7.96	0.22 ± 0.10	35.54 ± 12.46	67.62 ± 23.46	nd	nd	nd	nd
Sun-dried bream(n = 5) ^2^	15.94 ± 1.48	1.12 ± 0.63	110.60 ± 63.82	11.40 ± 8.74	1.17 ± 0.90	22.43 ± 17.32	55.80 ± 23.23	0.15 ± 0.07	nd	nd	nd
Sun-dried kapenta (n = 5) ^2^	17.67 ± 0.77	2.54 ± 0.56	187.80 ± 58.59	68.11 ± 2.59	2.84 ± 0.46	160.25 ± 14.43	33.60 ± 25.58	0.78 ± 0.03	nd	nd	nd
Fresh bream (n = 5) ^2^	15.39 ± 1.06	1.25 ± 1.14	163.00 ± 68.41	21.79 ± 12.32	1.55 ± 1.21	34.89 ± 18.82	54.00 ± 25.17	0.26 ± 0.16	nd	nd	0.06 ± 0.12
WHO and FAO		10.00	50.00	0.50	0.40–1.50	50.00		3.50	0.05–0.1	0.50–1.0	0.2–1.0
Reference		[26]	[26]		[27]	[27]		[26]	[28]	[28]	[28]

^1^first sampling 2021 and ^2^second sampling 2022, nd = not detected. n = number of samples

### Essential Trace Elements

Data obtained for Cr and Mn were above the acceptable limits set by FAO/WHO in all the samples. Cr was detected in all fish sampled with varying concentrations ranging from 15.47 to 19.53 mg/kg, in the sun-dried and fresh bream, respectively for the 2021 sampling period, whereas in the 2022 sampling period, the concentrations ranged from 15.39 to 17.67 mg/kg in the fresh bream and sun-dried kapenta, respectively. It was evident that the Cr was from the same source, as there was no statistically significant difference in obtained concentrations (*p* > 0.05). Notably, the Cr level was 15 times above the permissible limits (1 mg/kg) recommended by FAO/WHO in foodstuff (Table 2). Cr (III) helps to transport blood glucose from the bloodstream into the cells as energy [18], whereas, Cr (VI) has shown to be carcinogenic [30].

Zn was mostly below the FAO/WHO acceptable limits except in sun dried Kapenta in second sampling period (2022) where its concentration was above the limits. Fe was also higher than acceptable limits in all samples except for the fresh bream sample in the first sampling period (2021). It is important to note that essential trace elements are needed in minimum amounts but can cause adverse health effects if their intake is either too low (Zn and Fe deficiency) or too high (Zn and Fe toxicity) than expected [31]. According to Baki et al. [32], fish muscles retain the lowest elemental concentrations and are usually not considered an active site for elements to accumulate and biotransform. However, it is still important to have the fish muscles assessed.

Se and Cu levels obtained in the first sampling period (2021) were significantly lower (*p* < 0.05) than levels obtained in the second sampling period (2022) in sun-dried kapenta and bream. Se and Cu were not detected in sun-dried bream (2021) samples, whereas they were found in sun-dried kapenta (0.45 and 1.09 mg/kg) and fresh bream (0.22 and 0.61 mg/kg), respectively. In 2022 samples of Se and Cu were found in all sampled fish. For sun-dried bream (1.17 and 1.12 mg/kg), sun-dried Kapenta (2.84 and 2.54 mg/kg) and in fresh bream (1.55 and 1.25 mg/kg), respectively. Cu and Se mean concentrations were within the recommended permissible limits by FAO/WHO in all samples, except for Se (Table 2) in 2022 samples. Se and Cu are essential elements for better health at a certain amount but at large amounts Se for example, can lead to an increased risk of several diseases such as cancer, heart diseases, viral diseases and other conditions that involve an increased oxidative stress [33].

### Non-Essential Trace Elements

Al present in all samples (Table 2). It was obtained below the maximum recommended limits by FAO/WHO set at 100 mg/kg in foodstuffs in all samples except in sun-dried Kapenta. Al is known as an endocrine disruptor and a neurotoxic agent, as the element tends to accumulate in the brain [12, 14]. Several studies have correlated certain Al concentrations and different diseases such as the Alzheimer’s disease [34, 35]. Considering that the samples were wrapped by aluminum foil, the high levels of Al especially in 2021 fresh bream could possibly be due to contamination from aluminum foil.

The As in fish samples ranged from 0.03 mg/kg in sun-dried bream in the 2021 sampling period to 0.78 mg/kg in sun-dried kapenta in the 2022 sampling period. The mean concentrations of As were observed to be within the permissible limits. The presence of As could possibly be due to coal mining upstream or due to geology [13]. Moreover, the sun-drying of kapenta at an open space nearby busy roads and an unhygienic open market is another source of contamination [19]. The mean concentrations of As found in this study were below those reported by Nuapia and colleagues [19] (3.48–12.04 mg/kg) in fish sold in open markets in central Johannesburg, South Africa and Kinshasa, Democratic Republic of Congo (DRC).

As previously stated, Cd, Hg and Pb were not detected in all fish samples, except for Pb in fresh bream in the 2022 sampling period. The mean concentration of Pb (0.06 mg/kg) found in the fresh bream were higher than those reported by Simukoko et al. [13] with concentrations ranging from 0.004 to 0.009 mg/kg in Kariba bream.

### Correlation Among Elements in Fish in First and Second Sampling

**Fig. 2:**
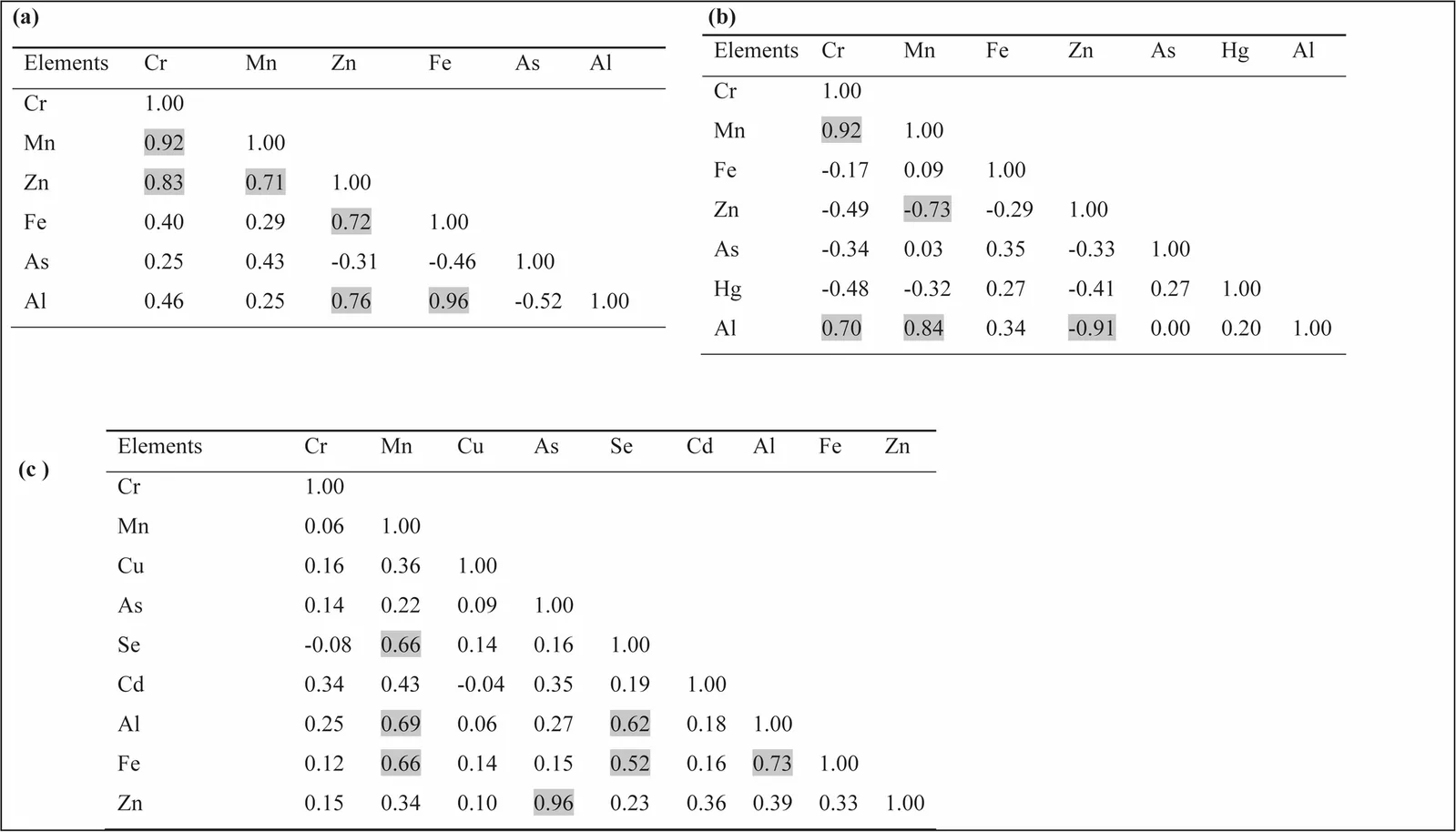
Correlation between trace elements in the 2021 sampling period for the different fish samples: (**a**) sun-dried bream, (**b**) sun-dried Kapenta and (**c**) fresh bream

**Fig. 3:**
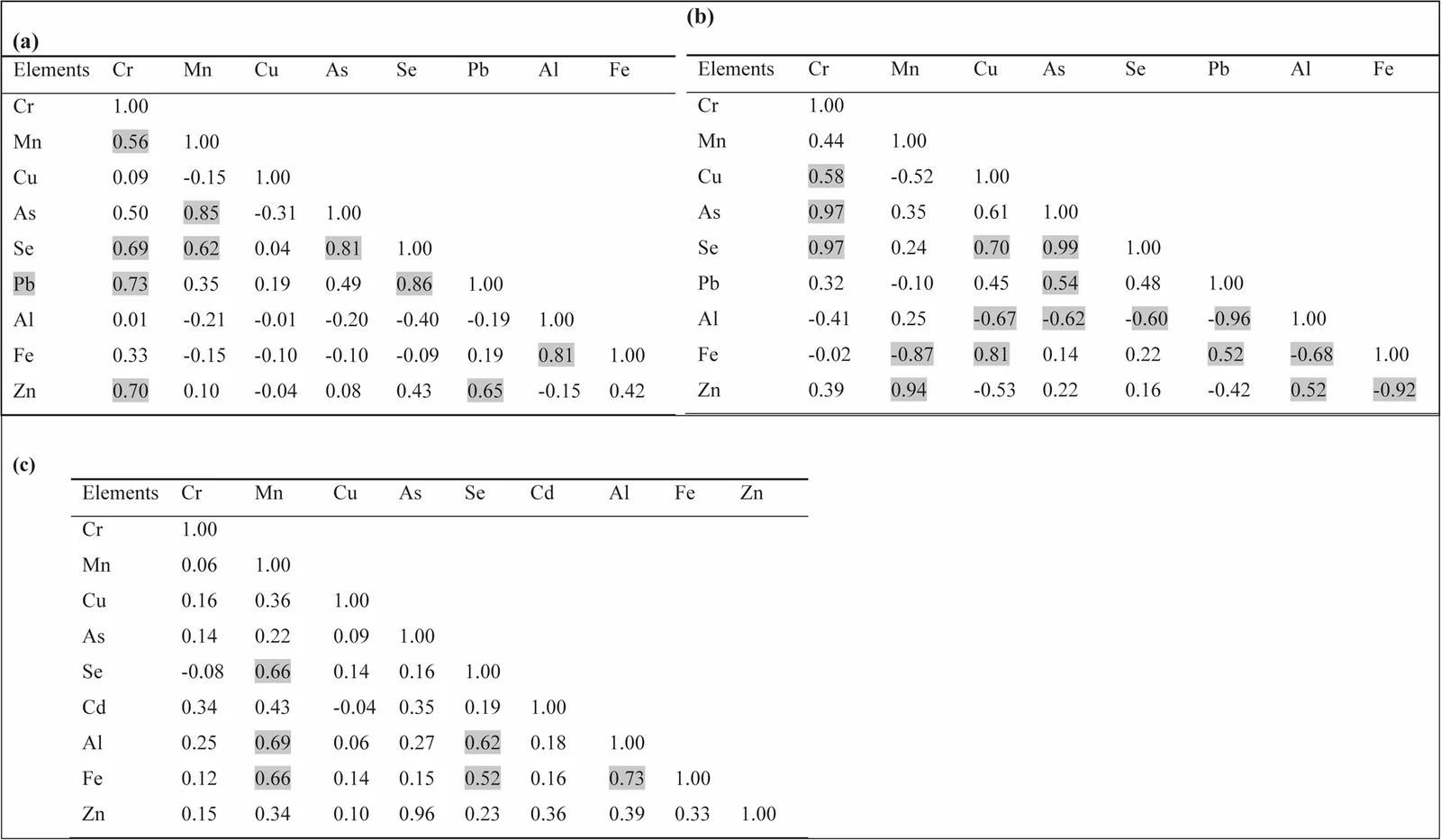
Correlation between trace elements in the 2022 sampling period for the different fish samples (**a**) sun-dried bream, (3**b**) sun-dried Kapenta, and (**c**) fresh bream

Correlation between target TEs obtained between the different fish species and with different seasons are shown in Figs. 2 and 3. Performing correlation analysis on the data enables the identification of possible common sources of trace elements in surface water that bioaccumulate into the fish [36]. There were no clear trends obtained in the correlation studies between seasons of the same type of fish and between the two types of fish species (Figs. 2 and 3). For example, in Fig. 2a&b comparison of correlation between sun dried bream and sun dried kapenta within same season shows that only Cr and Mn correlated positively in both fish types. Zn and Mn gave positive correlation in sundried bream but negative correlation in sun dried kapenta. The same trend is observed for Al and Zn. Comparing Figs. 2a and 3a of the sun-dried bream in two different seasons also not much same correlations are obtained except for Cr and Mn and Cr and Zn. The reason for this, especially between the two types of fish, could be due to trophic transfer differences in the food chain [13]. Other factors that include the changes in food composition, especially with a lot of activities happening within and around the lake, could have influenced the correlations between seasons [13]. In rainy season, one aspect various matrix composition being washed in the lake from land-based activities which have direct influence on food composition of the fish. Postharvest handling of the fish, especially open sun drying process of both kapenta and bream could have also played a role in trace elements contamination and correlation results [13].

### Comparison of Trace Element Concentrations in Fish Samples with Other Studies

A comparison of the TEs concentrations obtained in this study to other studies is shown in Table 3. Overall, the levels of trace elements in fresh Kariba bream were higher than those obtained by Simukoko et al. [13]. In this study, Cd, Pb and Hg were not detected in all fish sampled except for Pb in one fresh bream sample but also at trace level. Simukoko et al. [13] detected Hg in some samples but was found to be in trace amounts. Cr, Fe, Mn and Zn were generally found to be in higher concentrations than other studies shown in Table 3 including from that of Simukoko et al. [13]. A study by Nuapia et al. [19] of fish sold in open markets in Johannesburg, South Africa and Kinshasha, Democratic Republic of Congo somewhat had also slightly higher TE concentration and in some cases closer to what was obtained in this study (Table 3). This may suggest that post-handling of the fish from its habitat until it is sold in open markets may contribute to TE load.

**Table 3:** Comparison of mean concentration of trace elements (mg/kg) obtained in this study with other studies

Country	Sample type	Cr	Mn	Cu	As	Se	Cd	Al	Fe	Zn	Hg	Pb	References
Zambia	sun-dried bream	15.71	8.95	1.12	0.09	1.17	nd	66.12	111.95	16.25	nd	nd	This study
Zambia	sun-dried kapenta	17.57	51.48	2.01	0.63	1.50	nd	95.81	179.40	114.39	nd	nd	This study
Zambia	fresh bream	17.46	17.89	0.93	0.26	0.89	nd	60.81	128.96	35.22	nd	0.06	This study
Zambia	fresh bream	0.095	nd	0.25	0.022	0.152	0.001	4.81	5.23	4.76	0.021	0.009	[13]
Zambia	fresh bream	0.016	nd	0.33	0.042	0.176	0.002	4.28	5.34	4.75	0.008	0.009	[13]
Zambia	fresh bream	0.021	nd	2.14	0.023	0.135	0.001	0.78	8.16	8.49	0.009	0.004	[13]
Zambia	kapenta	Nd	nd	Nd	nd	nd	0.19	nd	Nd	nd	nd	0.44	[37]
Tanzania	fresh tilapia	Nd	nd	0.32	nd	nd	nd	nd	Nd	28.30	nd	0.32	[38]
Egypt	fresh tilapia	0.326	nd	0.11	0.367	nd	˂0.012	2.59	2.031	nd	nd	0.008	[39]
Egypt	fresh tilapia	Nd	nd	Nd	nd	nd	0.15	nd	Nd	nd	1.18	0.54	[17]
SA	fresh tilapia	0.36	10.51	6.53	3.48	0.12	0.64	9.10	Nd	14.14	nd	0.39	[19]
DRC	fresh tilapia	3.56	11.84	11.73	12	0.17	2.50	4.63	Nd	50.69	nd	1.54	[19]

*nd*  not detected, *DRC*  Democratic Republic of Congo, *SA*  South Africa

The presence of Cr, Al, Fe and Cu level was contrary to expectations to the number of Kapenta fishing boats that rose from 423 in 2009 to 962 in 2013; as these are thought to be the sources of Cr, Al, Fe and Cu [13, 35]. In this study, no Hg was detected in all fish samples, however, in the study by Simukoko et al. [13] in the past reported the presence of Hg in wild and farmed fish from lake Kariba with a range of 0.021, 0.008 and 0.009 mg/kg which was attributed to coal mining around the lake. Furthermore, a study by Hamada and coworkers [17] detected Hg (1.18 mg/kg) in tilapia. Essentially, more studies need to be done on mercury analysis, as it could bioaccumulate if ignored and not properly screened and determined. Research shows that food with Hg has adverse implications on human health and on the economy [2, 40].

### Health Risk Assessment

#### Estimated Weekly Intake (EWI), Target Hazard Quotient (THQ), Hazard Index and CR

**Fig. 4:**
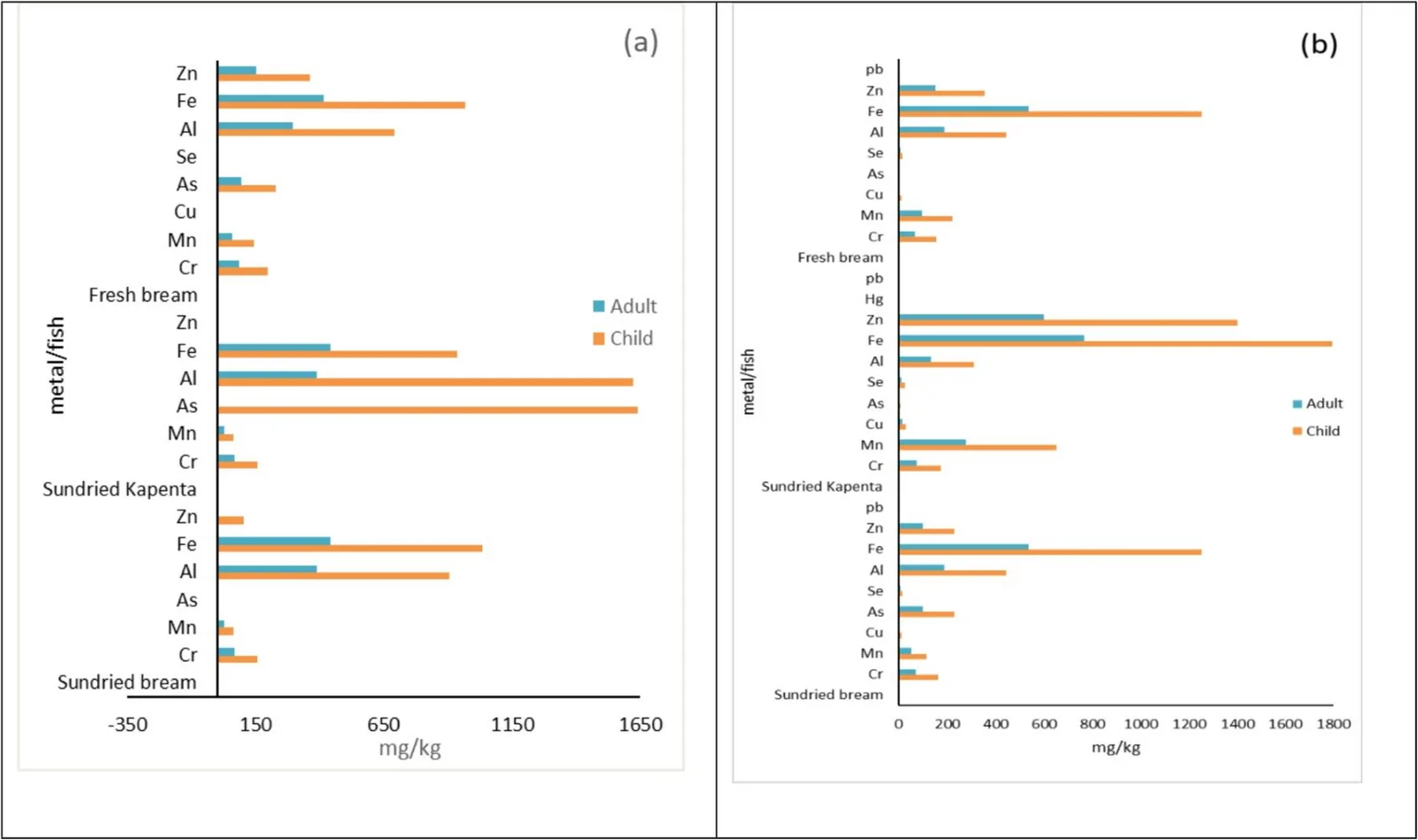
Health risk assessment for ingestion of fish from Lake Kariba (**a**) EWI ingestion for 2021 and (**b**) EWI ingestion for 2022

The estimated weekly intakes (EWI) values of Cr, Mn, Cu, As, Se, Cd, Al, Fe, Zn and Pb in fresh bream, sun-dried bream and sun-dried Kapenta for both adults and children are presented in Fig. 4 for both the 2021 and 2022 sampling periods. The EWI of the TEs for the 2021 sampling followed the order: Fe > Al > Zn > As > Cr > Mn > Cu > Cd > Se in fresh bream; Al > Fe > Zn > Mn > Cr > Cu > As > Se in sun-dried Kapenta and Fe > Al > Cr > Zn > Mn > As in sun-dried bream. The average EWI (2022) followed the order: Fe > Al > Zn > Cr > Mn > As > Cu > Se in fresh bream; Al > Fe > Zn > Mn > Cr > As in sun-dried Kapenta and Fe > Al > Zn > Cr > Mn > Se > Cu > Pb > As in sun-dried bream. A study by Simokoko and colleagues [13] obtained EWI values ranging from 1.1 × 10^− 6^ – 2.83 × 10^− 2^ for the target TEs, which is much lower than what was obtained in this study. Other studies like that of Munyeshuri et al. [8] calculated estimated daily intake (EDI) and obtained values ranging from 1.85 × 10^− 7^ to 2.39 × 10^− 2^, which is also lower than values from this study. However, EDI often is difficult to compare with other studies unless calculations are standardized. In our case it was obtained EWI divided by 7 days. The sundried kapenta for some metals showed highest estimated weekly intake. This further suggests that metal concentrations in the fish samples is influenced by several factors including postharvest handling and other environmental factors [13].

Table 4 demonstrates the calculated values for THQ and HI in sundried bream, whilst values for the sun-dried kapenta and fresh are presented in Tables 1S-2S. Notably, the THQ and HI for some elements were above the threshold of 1 (Tables 1S-4S) posing risk of non-carcinogenic to human health for long term consumption [1]. Moreover, the oral reference dose (RfD, mg/kg bw/day) for the target TEs is Al (1), Cr (0.003), Fe (0.7), Cu (0.04), Zn (0.3), As (0.0003), Se (0.005), Cd (0.001), Hg (0.0001), Pb (0.001) and Mn (0.14) [13].

**Table 4:** Health risk assessment on consumption of sun-dried bream from Lake Kariba, 2021 and 2022

Elements	Child		Adult	
	2021	2022	2021	2022
Cr	1.04 × 10^4^	1.06 × 10^4^	2.22 × 10^3^	2,29 × 10^3^
Mn	9.33 × 10^1^	1.64 × 10^2^	2.00 × 10^1^	3,51 × 10^1^
Cu	-	5.62 × 10^1^	-	1,21 × 10^1^
As	2.41 × 10^1^	9.71 × 10^1^	5.17	2,08 × 10^1^
Se	-	4.71 × 10^2^	-	1,01 × 10^1^
Al	1.78 × 10^2^	8.73 × 10^1^	3.82 × 10^1^	1,87 × 10^1^
Fe	2.91 × 10^2^	3.52 × 10^2^	6.24 × 10^1^	7,53 × 10^1^
Zn	6.73 × 10^1^	1.50 × 10^2^	1.44 × 10^1^	3,21 × 10^1^
Pb	-	6.77 × 10^2^	-	1,45 × 10^2^
HI	1.07 × 10^4^	1.27 × 10^4^	2.3 × 10^3^	2.63 × 10^3^

The calculated lifetime cancer risk (LCR) which is the health risk posed by carcinogenic metals was above the acceptable threshold value 1 × 10^− 4^ for most metals (Tables 3S-5S). The European Union and WHO has set an Environmental Quality Standard (EQS) of 0.02 and 0.5 mg kg^− 1^ of mercury in fish, respectively [13, 31]. In this regard, the fish analysed was safe from adverse health effects related to Hg implications as Hg was not detected in all fish samples (Table 1).

## Conclusion

This study monitored and assessed the total mean concentrations of various trace elements in fresh bream, sun-dried bream and Kapenta fish as well as human health risks associated with these trace elements. The samples were sampled in Siavonga, Lake Kariba’s most renowned Kariba bream and Kapenta producers. Metals like Fe, Mn and Cr in some fish samples from the open market contained higher concentrations above permissible limits proposed by the FAO/WHO. However, the findings indicate that fish do not present any discernible risk in consumption on human well-being. The high levels of some metals could be attributed to postharvest handling and processing. Thus, improvements in this area could be recommended by Local authority.

## Supplementary Information

Below is the link to the electronic supplementary material.

Supplementary Material 1

## Data Availability

Data generated can be provided on reasonable request.
