# Supplementary material for: Monitoring and Human Health Assessment of Trace Elements in Fresh and Dry Fish From Lake Kariba, Siavonga, Zambia
Source: Biol Trace Elem Res. 2026 Aug 5;204(11):8349–60. doi: 10.1007/s12011-026-05263-7 (PMC13714133; doi:10.1007/s12011-026-05263-7)
Supplement: Supplementary file 1 [file 12011_2026_5263_MOESM1_ESM.docx]

**Supplementary data**

**Monitoring and human health assessment of trace elements in fresh and dry fish from Lake Kariba, Siavonga, Zambia**

Biological Trace Element Research

Phaphedi M Poopedi^a,b^, Sinegugu Khulu^c^, Kawawa Banda^d^, Heidi Richards^b^, Mokgaetji Monyai^b^, Imasiku Nyambe^d^, Yoshitaka Uchida^e^, Luke Chimuka^b*^

^a^Department of Chemistry, Faculty of Science, Engineering and Agriculture, University of Venda, Private Bag X5050, Thohoyandou 0950, South Africa

^b^Molecular Sciences Institute, School of Chemistry, University of Witwatersrand, Private Bag X3, Johannesburg 2050, South Africa

^c^School of Education, University of Witwatersrand, 27 St. Andrews Rd, Parktown, Johannesburg, 2193, South Africa

^d^University of Zambia, Integrated Water Resources Management Centre, School of Mines, P.O. Box 32379, Lusaka, Zambia

^e^Research Faculty of Agriculture, Hokkaido University, Kita 9, Nishi 9, Kita-ku, Sapporo, Hokkaido 060-8589, Japan

* Corresponding author: [luke.chimuka@wits.ac.za](mailto:luke.chimuka@wits.ac.za); ORCID ID.: 0000-0002-8552-2478

**Hazard Quotient and Hazard Index**

| **Table 1S:** Health risk assessment on consumption of sun-dried Kapenta from Lake Kariba, 2021 and 2022 | | | | | |
| --- | --- | --- | --- | --- | --- |
|  | **Child** | | **Adult** | | |
|  | **2021** | **2022** | **2021** | **2022** | |
| **Elements** | **THQ_Ing_** | **THQ_Ing_** | **THQ_Ing_** | | **THQ_Ing_** |
| Cr | 1,2197E+04 | 1,1338E+04 | 2,2212E+03 | | 2,4296E+03 |
| Mn | 7,1429E+00 | 9,1384E+02 | 1,2081E+02 | | 1,9582E+02 |
| Cu | 2,5000E+01 | 1,4678E+02 | 1,1733E+01 | | 3,1453E+01 |
| As | 3,2685E+02 | 5,0769E+02 | 5,1669E+00 | | 1,0879E+02 |
| Se | 2,0000E+02 | 1,0215E+03 | 3,8751E+01 | | 2,1890E+02 |
| Pd | - | 7,8364E+02 | - | | 1,6792E+02 |
| Al | 3,2294E+02 | 6,2088E+01 | 3,8235E+01 | | 1,3305E+01 |
| Fe | 6,1369E+02 | 5,7352E+02 | 6,2371E+01 | | 1,2290E+02 |
| Zn | 4,5641E+02 | 9,1861E+02 | 1,4436E+01 | | 1,9685E+02 |
| HI | 9,8759E+05 | 1,03E+07 | 1,6916E+07 | | 2,2776E+07 |

| **Table 2S:** Health risk assessment on consumption of fresh bream from Lake Kariba, 2021 and 2022 | | | | |
| --- | --- | --- | --- | --- |
|  | Child | | Adult | |
|  | 2021 | 2022 | 2021 | 2022 |
| **Elements** | **THQ_Ing_** | **THQ_Ing_** | **THQ_Ing_** | **THQ_Ing_** |
| Cr | 1,3081E+04 | 1,0306E+04 | 2,8032E+03 | 2,2085E+03 |
| As | - | 1,7124E+02 | - | 3,6694E+01 |
| Pb | - | 1,1152E+02 | - | 2,3897E+01 |
| HI | 2,5660E+06 | 1,0442E+06 | 4,4087E+07 | 1,7940E+07 |

**Carcinogenic/cancer risk (CR) and the total cancer risk (TCR)**

| **Table 3S:** Health risk assessment of CR and TCR on consumption of sun-dried bream from Lake Kariba, 2021 and 2022 | | | | | | | | |
| --- | --- | --- | --- | --- | --- | --- | --- | --- |
|  | Child | | | | Adult | | | |
|  | 2021 | | 2022 | | 2021 | | 2022 | |
| **Elements** | **CR_Ing_** |  | **CRIng** |  | **CRIng** |  | **CRIng** |  |
| Cr | 1,5548E+01 |  | 1,6012E+01 |  | 3,3318E+00 |  | 3,4311E+00 |  |
| As | 1,0850E-01 |  | 4,3703E-01 |  | 2,3251E-02 |  | 9,3649E-02 |  |
| Pb | - |  | 5,7557E-03 |  | - |  | 1,2334E-03 |  |
| TCR | 1,4108E+03 | | 1,4827E+03 | | 2,4240E+04 | | 2,5475E+04 | |

| **Table 4S:** Health risk assessment of CR and TCR on consumption of sun-dried Kapenta from Lake Kariba, 2021 and 2022 | | | | | | | | | |
| --- | --- | --- | --- | --- | --- | --- | --- | --- | --- |
|  | Child | | | | Adult | | | | |
|  | 2021 | | 2022 | | 2021 | | 2022 | | |
| **Elements** | **CR_Ing_** |  | **CRIng** |  | **CRIng** |  | | **CRIng** |  |
| Cr | 1,829E+01 |  | 1,701E+01 |  | 3,920E+00 |  | | 3,644E+00 |  |
| As | 1,471E+00 |  | 2,285E+00 |  | 3,152E-01 |  | | 4,896E-01 |  |
| Pd | - |  | 6,661E-03 |  | - |  | | 1,427E-03 |  |
| TCR | 7,4794E+13 | | 1,1651E+14 | | 3,0602E+04 | | | 2,9878E+04 | |

| **Table 5S:** Health risk assessment of CR and TCR on consumption of fresh bream from Lake Kariba, 2021 and 2022* | | | | | | | | |
| --- | --- | --- | --- | --- | --- | --- | --- | --- |
|  | Child | | | | Adult | | | |
|  | 2021 | | 2022 | | 2021 | | 2022 | |
| **Elements** | **CR_Ing_** |  | **CRIng** |  | **CRIng** |  | **CRIng** |  |
| Cr | 1,9622E+01 |  | 1,5460E+01 |  | 4,2048E+00 |  | 3,3128E+00 |  |
| As | 6,7225E+01 |  | 7,7058E-01 |  | 1,4405E+01 |  | 1,6512E-01 |  |
| Pb | - |  | 9,4790E-04 |  | - |  | 2,0312E-04 |  |
| TCR | 9,8469E+03 | | 3,4846E+03 | | 1,3446E+05 | | 2,5129E+04 | |
